# The 15q11.2 BP1–BP2 Microdeletion Syndrome: A Review

**DOI:** 10.3390/ijms16024068

**Published:** 2015-02-13

**Authors:** Devin M. Cox, Merlin G. Butler

**Affiliations:** Departments of Psychiatry & Behavioral Sciences, University of Kansas Medical Center, 3901 Rainbow Boulevard, MS 4015, Kansas City, KS 66160, USA; E-Mail: mbutler4@kumc.edu

**Keywords:** 15q11.2 BP1–BP2 microdeletion, Burnside-Butler syndrome, clinical and behavioral phenotype, chromosome breakpoints BP1 and BP2, Prader-Willi and Angelman syndromes, language and motor delays, autism, review

## Abstract

Patients with the 15q11.2 BP1–BP2 microdeletion can present with developmental and language delay, neurobehavioral disturbances and psychiatric problems. Autism, seizures, schizophrenia and mild dysmorphic features are less commonly seen. The 15q11.2 BP1–BP2 microdeletion involving four genes (*i.e.*, *TUBGCP5*, *CYFIP1*, *NIPA1*, *NIPA2*) is emerging as a recognized syndrome with a prevalence ranging from 0.57%–1.27% of patients presenting for microarray analysis which is a two to four fold increase compared with controls. Review of clinical features from about 200 individuals were grouped into five categories and included developmental (73%) and speech (67%) delays; dysmorphic ears (46%) and palatal anomalies (46%); writing (60%) and reading (57%) difficulties, memory problems (60%) and verbal IQ scores ≤75 (50%); general behavioral problems, unspecified (55%) and abnormal brain imaging (43%). Other clinical features noted but not considered as common were seizures/epilepsy (26%), autism spectrum disorder (27%), attention deficit disorder (ADD)/attention deficit hyperactivity disorder (ADHD) (35%), schizophrenia/paranoid psychosis (20%) and motor delay (42%). Not all individuals with the deletion are clinically affected, yet the collection of findings appear to share biological pathways and presumed genetic mechanisms. Neuropsychiatric and behavior disturbances and mild dysmorphic features are associated with genomic imbalances of the 15q11.2 BP1–BP2 region, including microdeletions, but with an apparent incomplete penetrance and variable expressivity.

## 1. Introduction

Chromosome 15 contains five common breakpoint sites along the proximal long arm; they are commonly referred to as BP1–BP5. There is a cluster of low copy DNA repeats located within this chromosome region which can facilitate mis-alignment during meiosis leading to non-allelic homologous recombination [1,2]. These low copy repeat sequences are called duplicons and contain pseudogenes [3]. Duplicons found within breakpoints BP1, BP2 and BP3 have been characterized by the presence of the *HERC2* gene (at BP3) and *HERC2* pseudogenes (at BP1 and BP2) [2].

**Figure 1:**
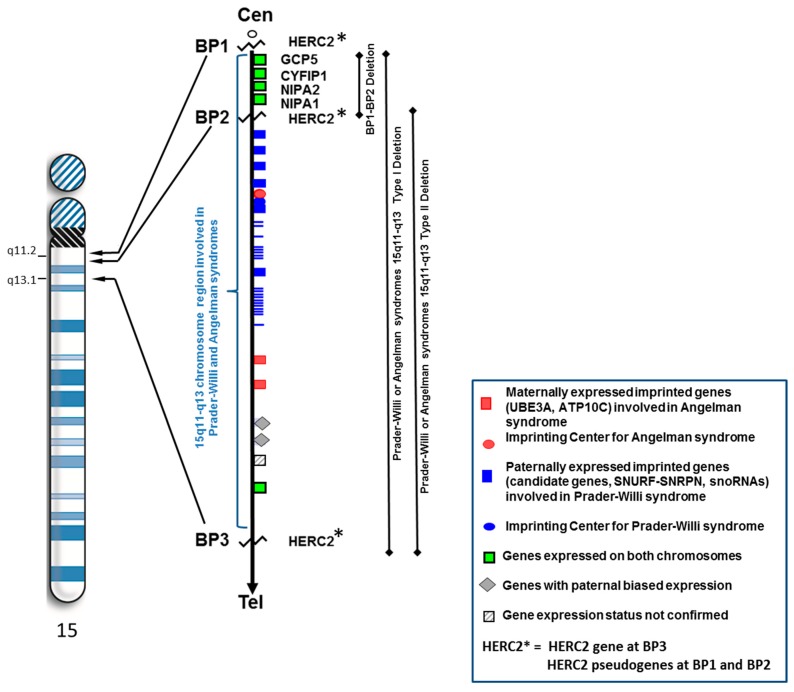
High resolution ideogram representing chromosome 15 showing location of breakpoints BP1 and BP2 (at 15q11.2 band) and BP3 (at 15q13.1 band) involving HERC2 and position of the non-imprinted genes between BP1 and BP2. The three deletion types involving the 15q11–q13 region (*i.e*., BP1–BP2, typical type I, typical type II) are represented.

Prader-Willi syndrome (PWS) and Angelman syndrome are typically caused by a deletion of different parental origin involving the distal breakpoint BP3 and proximally placed breakpoints BP1 or BP2. These cytogenetic deletions of chromosome 15q11–q13 region are classified as typical type I (involving BP1 and BP3) or typical type II (involving BP2 and BP3) (see Figure 1). Type I deletions have an average genomic length of 6.58 Mb while type II deletions have a mean length of 5.33 Mb [4]. Several studies have shown that individuals with the larger typical 15q11–q13 type I deletion which is found in both Prader-Willi and Angelman syndromes are reported to have more severe neurodevelopmental symptoms as compared to those individuals with the smaller typical type II deletion [5,6,7,8,9,10]. Initially, Butler *et al.* [5] found that several behavioral and intelligence measures were statistically different between the two PWS deletion types (type I and type II). PWS individuals with type I deletions showed more compulsive and self-injurious behaviors and visual perception impairment along with lower intelligence, reading and math scores than in those with type II deletions. Furthermore, Bittel *et al.* [6] reported that the amount of mRNA isolated from lymphoblastoid cell lines established from individuals with PWS for the four genes (*i.e*., *NIPA1*, *NIPA2*, *CYFIP1*, *TUBGCP5*) found in the genomic area between BP1 and BP2 in the 15q11.2 chromosome band explained between 24% to 99% of the phenotypic variability in behavioral and academic measures. The *NIPA2* gene accounted for the largest number of significant correlations between the mRNA levels and phenotypic features.

In other studies, Varela *et al.* [10] found that individuals with PWS having 15q11–q13 type I deletions acquired speech later than those with type II deletions. Hartley *et al.* [7] also found that individuals with PWS having type I deletions had significantly higher Reiss maladaptive behavior scores for depression (physical signs) than individuals with type II deletions. Similarly, Sahoo *et al.* [9] reported that in individuals with Angelman syndrome and the 15q11–q13 type I deletion had significantly more behavioral and cognitive impairments with lower expressive and total language abilities and a higher likelihood of features for autism spectrum disorder. 

Valente *et al.* [11] also reported in Angelman syndrome that those with the 15q11–q13 type I deletion had more severe seizures and were refractory to treatment compared with those having the type II deletion. In a separate study Milner *et al.* [8] found significantly higher verbal IQ scores in individuals with PWS and type II deletions *versus* those with type I deletions. They further reported that those with type I deletions performed more poorly on all measures of ability even though they were not significantly different from the patients with type II deletions. 

A report by Dykens and Roof [12] examined behaviors in PWS using a mixed cohort of young and old subjects (*n* = 88) and showed a relationship between genetic subtypes and ages. They found negative associations between age and behavior in the 15q11–q13 type I deletion subtype only which implicated non-imprinted genes between breakpoints BP1 and BP2, specifically the *CYFIP1* gene. Disturbed expression of *CYFIP1* is seen in other developmental disabilities including those with 15q disorders without PWS. Although they reported no significant behavioral findings when combining data from subjects at all ages, significant differences were found in those with type I *versus* type II deletions with age. The individuals with the 15q11–q13 type I deletion consistently had lower targeted problem behaviors and adaptive skills and externalizing symptoms with advancing age.

As several studies have shown evidence of disturbed gene expression patterns and behavioral findings in subjects with either PWS or Angelman syndrome with different genetic deletion subtypes implicating genes within the BP1 and BP2 genomic region, Burnside *et al.* [13] summarized the literature and surveyed the first large cohort of patients presenting for genetic testing using high resolution microarrays. They found that 0.86% of the approximate 17,000 individuals had an abnormality (deletion or duplication) of the 15q11.2 BP1–BP2 region. Specifically, 69 subjects were found with the 15q11.2 microdeletion and 77 subjects were found with a microduplication of the same region. They proposed that this genomic area was a susceptibility region for neurological dysfunction, impaired development and characteristic phenotypic features which was initially raised by Butler *et al.* [5] in a study of PWS individuals with the larger 15q11–q13 type I deletion including the four genes in the BP1 and BP2 area and having a more severe behavior phenotype compared with those with the smaller type II deletion. Collectively, they summarized that a deletion involving this genomic region correlated with language or motor delays, behavioral problems, autism, seizures and occasionally mild dysmorphic features. The collection of clinical findings in the microdeletion in a large cohort of patients with the 15q11.2 BP1–BP2 presenting for genetic services supported the original observations by Butler *et al.* [14] of behavioral disturbances seen in PWS patients with the larger 15q11–q13 type I deletion compared with the smaller type II deletion which stimulated interest in additional studies of this chromosome region and, hence, coined the Burnside-Butler syndrome. 

Preliminary clinical information about the 15q11.2 microdeletion alone without PWS was first reported by Murthy *et al.* [15] in 2007 in two individuals in a consanguineous family and later by Doornbos *et al.* [16] in 2009 in nine individuals. The vast majority of their combined subjects presented with behavioral or neurological problems. Later, Abdelmoity *et al.* [17] reported a cohort of 1654 consecutive pediatric patients presenting with a range of neurological disorders and found that 21% or 1.27% of the patients carried a 15q11.2 BP1–BP2 deletion. They found that 87.5% of the patients with the deletion had developmental delay or intellectual disability. More recently, Cafferkey *et al.* [18] presented data from 14,605 patients (primarily pediatric) referred for genetic testing using microarray analysis and found 83 (0.57%) with the 15q11.2 BP1–BP2 microdeletion. The majority of their patients presented with some form of behavioral disturbance or developmental/motor delays as summarized by Burnside *et al.* [13]. The area between BP1 and BP2 is approximately 500 kb in size and includes the *NIPA1*, *NIPA2*, *TUBGCP5*, and *CYFIP1* genes and prone to both microdeletions and microduplications. In this review, we summarize information regarding the 15q11.2 BP1–BP2 microdeletion. A review of information regarding the microduplication is beyond the scope of this report. 

Chai *et al.* [19] showed that these four genes are highly conserved and biallelically expressed. *NIPA1* or non-imprinted in Prader-Willi/Angelman syndrome 1 gene is the best studied gene within this region and associated with autosomal dominant hereditary spastic paraplegia [20,21]. There has been no case to date where haploinsufficiency of *NIPA1* due to a deletion has led to hereditary spastic paraplegia. *NIPA1* also mediates Mg^2+^ transport and is highly expressed in neuronal tissue [22]. The *NIPA2* or non-imprinted in Prader-Willi/Angelman syndrome 2 gene is used in renal Mg^2+^ transport [22]. Jiang *et al.* [23] examined patients with childhood absence epilepsy and found mutations in *NIPA2* with unknown functional effects. The *TUBGCP5* gene or tubulin gamma complex associated protein 5 gene is involved in neurobehavioral disorders including ADHD and OCD [24]. The final gene within this region is *CYFIP1* or cytoplasmic fragile X mental retardation 1 (FMR1) interacting protein 1 gene. This gene product interacts with FMRP in a ribonucleoprotein complex. FMRP is the product of the *FMR1* gene which is associated with fragile X syndrome, the most common cause of familial intellectual disability that primarily affects males [25]. Both of these gene products play important roles in the regulation of brain mRNAs [24]. Bozdagi *et al.* [26] showed that haploinsufficiency of *CYFIP1* resembles important aspects found in knockout FMR1 mice. 

Not all individuals with defects within the 15q11.2 band (*i.e*., microdeletions or microduplications) share a clinical phenotype or are clinically affected. Therefore, this region contains genetic material showing incomplete penetrance or low penetrance of pathogenicity along with variable expressivity. For example, a review of reported data from control cohorts (*n* = 66,462 subjects) summarized to date show that about 0.25% of controls are found with the 15q11.2 BP1–BP2 microdeletion [18,27,28,29,30,31]. The penetrance of the 15q11.2 BP1–BP2 microdeletion has also been estimated at 10.4% [28] or an approximate two fold increase over the general population risk but may be due to paucity of inheritance data. Other estimates have been lower but results consistently show this region to play a role in autism [32]. The penetrance for the 15q11.2 microdeletion is low compared to other microdeletion syndromes such as the 16p11.2 deletion with a penetrance estimated at 62.4% [28]. A higher penetrance is often seen in copy number variants (CNVs) that have higher *de novo* frequencies while low penetrance estimates may reflect a subclinical presentation or manifestation of features that are recognized as components of disorders such as neuropsychiatric disturbances in parents of affected individuals (e.g., autism in 15q11.2 BP1–BP2 deletion) or in control cohorts. Not only are microarray studies needed on other family members (and parents) of those with the 15q11.2 BP1–BP2 deletion but also neuropsychiatric and behavioral testing as well to appreciate the variability of expression and level of penetrance. The review of literature from six published reports summarized by Cafferkey *et al.* [18] regarding information on the inheritance of the 15q11.2 BP1–BP2 microdeletion indicates that 22/43 (51%) of individuals with the microdeletion, where parental data were available, inherited their deletion from an apparently healthy parent while 10/29 (35%) of individuals inherited their deletion from an abnormal parent [13,15,16,17,33,34]. The phenotypic information was unavailable or incomplete for all parents found to carry the deletion. The reported *de novo* deletion frequency ranged from 1/21 (5%) [13] to 2/9 (22%) [16] in subjects reviewed by Cafferkey *et al.* [18]. It would be of importance to undertake DNA sequencing analyses of the 15q11.2 BP1–BP2 genomic region to identify subtle deletions or mutations of the non-deleted allele and determine the genetic status of this “normal allele”. Other modifying genes outside of the chromosome region may also play a role and will require further investigations. 

Recently, individuals have been reported with the 15q11.2 BP1–BP2 microdeletion and additional non-neurological clinical findings. These findings included congenital cataracts [14,35], proximal esophageal atresia and distal tracheoesophageal fistula (type C) [35] and congenital arthrogryposis [36]. These clinical reports lend support for further phenotypic expansion of this susceptibility region impacted by the 15q11.2 BP1–BP2 microdeletion. Our report will focus on the review of the clinical features now recognized in this microdeletion syndrome. 

## 2. Results and Discussion

### 2.1. Growth and Development 

A review of the literature on growth and development in individuals with 15q11.2 BP1–BP2 microdeletions involving a susceptibility region for neurological dysfunction with general developmental and motor delay with speech problems as primary components of this disorder. General developmental delay occurred in 73% of individuals and speech delay occurred in 67% as reported in the literature suggesting characteristic features for this microdeletion syndrome (see Table 1). These features may correlate with the neuronal components of the genes within this deleted region. *NIPA1*, *NIPA2*, and *CYFIP1* are highly expressed in neuronal tissue of the central nervous system and *TUBGCP5* is highly expressed in the subthalamic nuclei [22,24]. 

Motor delay and microcephaly were considered important features of this microdeletion syndrome after a review of the literature. Motor delay was seen in 42% of individuals and microcephaly was seen in 24%. Occasional features found were intrauterine growth retardation (18%), short stature (10%), and macrocephaly (8%) (see Table 1).

### 2.2. Dysmorphic Features

A review of the literature on the dysmorphic features for those individuals with the 15q11.2 BP1–BP2 microdeletion or Burnside-Butler syndrome showed that this disorder does not appear to have a clear dysmorphic phenotype (see Table 2). This is supported by studies by Butler *et al.* [5] in examining individuals with Prader-Willi syndrome with 15q11–q13 type I deletion or type II deletion and observing behavioral and cognitive differences but not in growth parameters or other clinical findings indicating that the four genes present within the BP1–BP2 genomic area and when disturbed can lead to neurological or behavioral outcomes. 

### 2.3 Intelligence and Academic Achievement

General, non-specified, dysmorphic features were seen in 55 out of 141 individuals or 39% of those reported within the literature. The only common dysmorphic feature found from a review of the literature was palatal abnormalities with 46% of individuals being reported with these findings. Occasional dysmorphic features included broad forehead (21%), hypertelorism (18%), slender fingers (15%), pectus excavatum (13%), plagiocephaly (10%), dysmorphic nose (8%), dysmorphic teeth (8%), and contractures/arthrogryposis (8%). Reported dysmorphic features that, at present, are uncommon or not associated with this syndrome based on a literature review and include short fingers (5%), long narrow face (3%), small face (3%), and hypotelorism (3%) (see Table 2).

**Table 1:** Literature Review of Growth and Development for Individuals with Chromosome 15q11.2 BP1–BP2 Microdeletion Syndrome.

Feature	Murthy *et al.* [15]	Doornbos *et al.* [16]	Von der Lippe *et al.* [34]	Burnside *et al.* [13]	Abdelmoity *et al.* [17]	Madrigal *et al.* [33]	Wong *et al.* [35]	Cafferkey *et al.* [18]	Usrey *et al.* [36]	Rudd *et al.* [37]	Jerkovich & Butler [14]	Total (%)
IUGR ^1^	0/1	3/9	0/5	N/A	N/A	N/A	1/2	N/A	0/2	0/2	0/1	4/22 (18)
Short stature	0/1	1/9	1/5	N/A	N/A	N/A	0/2	N/A	0/2	N/A	0/1	2/20 (10)
Microcephaly	0/1	1/9	0/5	N/A	4/16	2/2	0/2	N/A	1/2	N/A	1/1	9/38 (24)
Macrocephaly	0/1	0/9	1/5	N/A	2/16	0/2	0/2	N/A	0/2	N/A	0/1	3/38 (8)
**Developmental delay (general)**	2/2	7/8	4/7	33/56	13/15 *	2/2	0/2	65/77	N/A	0/2	0/1	**126/172 (73)**
Motor delay	1/2	8/9	5/7	20/56	N/A	2/2	1/2	29/77	N/A	0/2	0/1	66/158 (42)
**Speech delay**	2/2	8/8	5/5	44/49	N/A	2/2	0/2	37/77	N/A	0/2	1/1	**99/148 (67)**

^1^ Intrauterine growth retardation; * indicated both Intellectual disability or Global developmental delay; Bold indicate categories that represent 50% or greater incidence within the literature.

**Table 2:** Literature Review of Dysmorphic Features for Individuals with Chromosome 15q11.2 BP1–BP2 Microdeletion Syndrome.

Feature	Doornbos *et al.* [16]	Von der Lippe *et al.* [34]	Burnside *et al.* [13]	Abdelmoity *et al.* [17]	Madrigal *et al.* [33]	Wong *et al.* [35]	Cafferkey *et al.* [18]	Usrey *et al.* [36]	Rudd *et al.* [37]	Jerkovich & Butler [14]	Total (%)
Dysmorphism, unspecified	N/A	N/A	27/56	N/A	N/A	N/A	28/83	N/A	0/2	N/A	55/141 (39)
Plagiocephaly	4/9	0/7	N/A	0/16	0/2	0/2	N/A	0/2	N/A	0/1	4/39 (10)
Broad forehead	5/9	0/7	N/A	1/16	2/2	0/2	N/A	0/2	N/A	0/1	8/39 (21)
Long narrow face	0/9	1/7	N/A	0/16	0/2	0/2	N/A	0/2	N/A	0/1	1/39 (3)
Small face	0/9	1/7	N/A	0/16	0/2	0/2	N/A	0/2	N/A	0/1	1/39 (3)
Hypertelorism	5/9	1/7	N/A	1/16	0/2	0/2	N/A	0/2	N/A	0/1	7/39 (18)
Hypotelorism	0/9	1/7	N/A	0/16	0/2	0/2	N/A	0/2	N/A	0/1	1/39 (3)
Abnormal nose	0/9	2/7	N/A	1/16	0/2	0/2	N/A	0/2	N/A	0/1	3/39 (8)
Dysmorphic ears	6/9	0/7	N/A	1/16	2/2	0/2	N/A	0/2	N/A	1/1	9/39 (46)
Palatal abnormalities	4/9	0/7	N/A	2/16	2/2	1/2	N/A	0/2	N/A	0/1	9/39 (46)
Abnormal teeth	0/9	2/7	N/A	1/16	0/2	0/2	N/A	0/2	N/A	0/1	3/39 (8)
Pectus excavatum	2/9	0/7	N/A	1/16	0/2	1/2	N/A	0/2	N/A	1/1	5/39 (13)
Contractures/arthrogryposis	0/9	1/7	N/A	0/16	0/2	0/2	N/A	2/2	N/A	0/1	3/39 (8)
Short fingers	0/9	1/7	N/A	1/16	0/2	0/2	N/A	0/2	N/A	0/1	2/39 (5)
Slender fingers	5/9	1/7	N/A	0/16	0/2	0/2	N/A	0/2	N/A	0/1	6/39 (15)

Intelligence and academic achievement for individuals with 15q11.2 BP1–BP2 microdeletions were reviewed (see Table 3). Common reported features included writing difficulties (60%), memory problems (60%), reading difficulties (57%), and a verbal IQ score equal to or below 75 (50%). However, very few individuals were reported within the literature regarding these findings so further studies are needed to determine if they are associated findings. Occasional findings within the literature included intellectual disability (37%) and performance IQ which was equal to or below 75 (33%). There were 43 out of 116 individuals reported within the literature who had intellectual disability; however, there were only 1 out of 3 individuals reportedly with a low performance IQ. Stefansson *et al.* [38] found a modest effect on verbal IQ (effect = 0.38, *p* = 0.033) and performance IQ (effect = 0.43, *p* = 0.018) from the 15q11.2 deletion.

### 2.4. Behavioral and Psychiatric Problems

Behavioral features including autism spectrum disorder and schizophrenia have been studied as features of the 15q11.2 BP1–BP2 microdeletion [32,33,37,38,39,40]. A review of the behavioral and psychiatric features reported within the literature found that 55% of those reported had general behavior problems that were not otherwise specified (see Table 4). Common behavioral features included Attention Deficit Disorder or Attention Deficit Hyperactivity Disorder (35%), Autism Spectrum Disorder (27%), obsessive compulsive disorder (26%), self-injurious behaviors (26%), oppositional defiant disorder (24%), and schizophrenia or paranoid psychosis (20%). Studies have been performed to determine if the 15q11.2 BP1–BP2 microdeletion is a susceptibility locus for schizophrenia [37,38,39,40]. Rees *et al.* [40] found a significant association between the microdeletion and schizophrenia with the frequency of individuals having schizophrenia and the microdeletion from their study was 0.64 (44/6882). The 15q11.2 BP1–BP2 microdeletion has also been studied regarding potential susceptibility for autism [32]. Chaste *et al.* [32] found a small risk regarding this microdeletion and autism but a greater effect on the autism phenotype in males and when maternally inherited. They reported an overall modest effect for this copy number variant for autism in studying a sample of 2525 families with autism [32]. They suggested that carriers may need additional copy number variants along with the 15q11.2 microdeletion in order to be diagnosed with autism [32]. Occasionally, reported behavioral features within the literature included an unusually happy expression (12%) and anxiety (6%) [16,34].

### 2.5. Other Related Medical Concerns

The 15q11.2 BP1–BP2 microdeletion or Burnside-Butler syndrome has been reported with various medical concerns or conditions (see Table 5). A review of the literature indicated that 43% of these individuals had abnormal brain imaging (MRI, EEG, *etc.*) with common clinical features of seizures or epilepsy (26%) and ataxia or balance (coordination) problems (28%). The medical literature surrounding this microdeletion syndrome and seizures indicate that this chromosomal anomaly may be a risk factor for epilepsy [29,41,42,43]. Occasional features seen within the literature regarding medical conditions included congenital heart defect (9%), genital abnormalities (7%), recurrent infections (7%), cataracts (4%), hearing loss or impairment (4%), tracheoesophageal fistula (2%), and omphalocele (2%). Whether these additional findings represent further phenotypic expansion of the syndrome as raised by others is unknown [14,35,36].

**Table 3:** Literature Review of Intelligence and Academic Achievement for Individuals with Chromosome 15q11.2 BP1–BP2 Microdeletion Syndrome.

Feature	Murthy *et al.* [15]	Doornbos *et al.* [16]	De Kovel *et al.* [29]	Von der Lippe *et al.* [34]	Burnside *et al.* [13]	Abdelmoity *et al.* [17]	Madrigal *et al.* [33]	Mullen *et al.* [43]	Jahn *et al.* [42]	Usrey *et al.* [36]	Rudd *et al.* [37]	Jerkovich & Butler [14]	Total (%)
ID */FSIQ ^1^ ≤ 75/special education	2/2	6/12	0/11	5/11	11/49	13/15	2/2	1/6	2/3	1/1	0/3	0/1	43/116 (37)
**Verbal IQ ≤ 75**	N/A	1/1	N/A	N/A	N/A	N/A	N/A	N/A	N/A	N/A	1/3	N/A	**2/4 (50)**
Performance IQ ≤ 75	N/A	N/A	N/A	N/A	N/A	N/A	N/A	N/A	N/A	N/A	1/3	N/A	1/3 (33)
**Reading difficulties**	N/A	N/A	N/A	4/7	N/A	N/A	N/A	N/A	N/A	N/A	N/A	N/A	**4/7 (57)**
**Writing difficulties**	N/A	N/A	N/A	3/5	N/A	N/A	N/A	N/A	N/A	N/A	N/A	N/A	**3/5 (60)**
**Memory problems**	N/A	N/A	N/A	2/4	N/A	N/A	N/A	N/A	N/A	N/A	N/A	1/1	**3/5 (60)**

* Intellectual disability; ^1^ Full scale intelligence quotient; Bold indicate categories that represent 50% or greater incidence within the literature.

**Table 4:** Literature Review of Behavioral and Psychiatric Problems for Individuals with Chromosome 15q11.2 BP1–BP2 Microdeletion Syndrome.

Feature	Murthy *et al.* [15]	Doornbos *et al.* [16]	Von der Lippe *et al.* [34]	Burnside *et al.* [13]	Abdelmoity *et al.* [17]	Madrigal *et al.* [33]	Cafferkey *et al.* [18]	Rudd *et al.* [37]	Jerkovich & Butler [14]	Total (%)
**General behavior problems, unspecified**	2/2	N/A	N/A	35/56	N/A	2/4	35/73	N/A	1/1	**75/136 (55)**
Autism Spectrum Disorder	1/2	4/9	1/7	14/49	2/16	2/4	19/73	N/A	0/1	43/161 (27)
Schizophrenia/paranoid psychosis	N/A	0/9	1/7	N/A	N/A	N/A	N/A	3/3	0/1	4/20 (20)
OCD ^1^	0/2	2/9	0/7	16/49 *	N/A	N/A	N/A	N/A	0/1	18/68 (26)
ODD ^2^	0/2	0/9	0/7	16/49 *	N/A	N/A	N/A	N/A	0/1	16/68 (24)
ADD ^3^/ADHD ^4^	2/2	2/9	0/7	16/49	7/12	N/A	N/A	N/A	1/1	28/80 (35)
Self-injurious behaviors	0/2	2/9	0/7	16/49 *	N/A	N/A	N/A	N/A	0/1	18/68 (26)
Anxiety	N/A	0/9	1/7	N/A	N/A	N/A	N/A	N/A	0/1	1/17 (6)
Happy expression	N/A	2/9	0/7	N/A	N/A	N/A	N/A	N/A	0/1	2/17 (12)

^1^ Obsessive compulsive disorder; ^2^ Oppositional defiant disorder; ^3^ Attention deficit disorder; ^4^ Attention deficit hyperactivity disorder; * indicates obsessive compulsive disorder, oppositional defiant disorder, self-injury, tantrums, *etc*; Bold indicate categories that represent 50% or greater incidence within the literature.

**Table 5:** Literature Review of Other Related Medical Concerns for Individuals with Chromosome 15q11.2 BP1–BP2 Microdeletion Syndrome.

Feature	Murthy *et al.* [15]	Doornbos *et al.* [16]	De Kovel *et al.* [29]	Von der Lippe *et al.* [34]	Burnside *et al.* [13]	Abdelmoity *et al.* [17]	Madrigal *et al.* [33]	Wong *et al.* [35]	Mullen *et al.* [43]	Cafferkey *et al.* [18]	Jahn *et al.* [42]	Usrey *et al.* [36]	Rudd *et al.* [37]	Jerkovich & Butler [14]	Total (%)
Seizures/epilepsy	0/2	2/8	8/23	0/7	14/56	2/16	0/2	0/2	6/6	13/83	4/5	1/2	0/3	0/1	57/216 (26)
Cataracts	0/1	0/9	N/A	0/7	N/A	0/16	0/2	1/2	N/A	N/A	0/3	0/2	0/3	1/1	2/46 (4)
Congenital heart defect	0/1	2/9	N/A	0/7	N/A	0/16	0/2	2/2	N/A	N/A	0/3	0/2	0/3	0/1	4/46 (9)
Genital abnormalities	0/1	2/9	N/A	1/7	N/A	0/16	0/2	0/2	N/A	N/A	0/3	0/2	N/A	0/1	3/46 (7)
Recurrent infections	0/1	2/9	N/A	0/7	N/A	N/A	0/2	0/2	N/A	N/A	0/3	0/2	N/A	0/1	2/30 (7)
Ataxia/balance issues	1/2	4/8	N/A	0/7	15/49	N/A	2/2	0/2	N/A	N/A	0/3	0/1	0/3	0/1	22/78 (28)
TE fistula	0/1	0/9	N/A	0/7	N/A	0/16	0/2	1/2	N/A	N/A	0/3	0/2	0/3	0/1	1/49 (2)
Hearing loss/impairment	0/1	1/9	N/A	0/7	N/A	1/16	0/2	0/2	N/A	N/A	0/3	0/2	0/3	0/1	2/49 (4)
Omphalocele	0/1	1/9	N/A	0/7	N/A	0/16	0/2	0/2	N/A	N/A	0/3	0/2	0/3	0/1	1/49 (2)
Abnormal brain imaging	N/A	0/4	N/A	1/2	20/56 *	2/3	N/A	N/A	N/A	N/A	5/5	1/2	3/3	N/A	32/75 (43)

* indicates cases with insomnia, abnormal brain MRI or EEG, *etc*.

## 3. Conclusions

The 15q11.2 BP1–BP2 microdeletion (Burnside-Butler) syndrome is now a recognized condition with over 200 individuals identified from the literature using chromosomal microarray analysis. Clinically, neurological dysfunction, developmental and language delay are the most commonly associated findings followed by motor delay, ADD/ADHD and autism spectrum disorder showing incomplete penetrance and variable expressivity. The four non-imprinted biallelically expressed genes (*TUBGCP5*, *CFYIP1*, *NIPA1*, *NIPA2*) in this microdeletion were initially noted to impact severity of clinical presentation and neurological impairment in two classical genomic imprinting disorders (*i.e*., Prader-Willi and Angelman syndromes) with typical 15q11–q13 deletions depending on the absence or presence of the genomic area between breakpoints BP1 and BP2 containing the four genes leading to studies recognizing this syndrome. The 15q11.2 BP1–BP2 microdeletion syndrome has a reported *de novo* frequency between 5%–22%, with 51% having inherited the microdeletion from an apparently unaffected parent and 35% having inherited the microdeletion from an affected parent. Low penetrance estimates may relate to subclinical manifestations of neuropsychiatric/behavioral problems or incomplete information about the parents of individuals with 15q11.2 BP1–BP2 microdeletion or members of control cohorts [44]. From reported patient cohorts presenting for genetic services and microarray analysis, this microdeletion syndrome can now be recognized as the most common cytogenetic abnormality found in autism spectrum disorder.
